# Quantification of Carbon Nanotube Doses in Adherent Cell Culture Assays Using UV-VIS-NIR Spectroscopy

**DOI:** 10.3390/nano9121765

**Published:** 2019-12-11

**Authors:** Dedy Septiadi, Laura Rodriguez-Lorenzo, Sandor Balog, Miguel Spuch-Calvar, Giovanni Spiaggia, Patricia Taladriz-Blanco, Hana Barosova, Savvina Chortarea, Martin J. D. Clift, Justin Teeguarden, Monita Sharma, Alke Petri-Fink, Barbara Rothen-Rutishauser

**Affiliations:** 1 Adolphe Merkle Institute, University of Fribourg, Chemin des Verdiers 4, 1700 Fribourg, Switzerland; dedy.septiadi@unifr.ch (D.S.); laura.rodriguez-lorenzo@inl.int (L.R.-L.); sandor.balog@unifr.ch (S.B.); miguel.spuch-calvar@unifr.ch (M.S.-C.); giovanni.spiaggia@unifr.ch (G.S.); patricia.taladrizblanco@unifr.ch (P.T.-B.); hana.barosova@unifr.ch (H.B.); savvina.chortarea@empa.ch (S.C.); 2 Department of Life Sciences, Nano for Environment Unit, Water Quality Group, Av. Mestre José Veiga s/n, 4715-330 Braga, Portugal; 3 In Vitro Toxicology Group, Swansea University Medical School, Swansea SA2 8PP, Wales, UK; m.j.d.clift@swansea.ac.uk; 4 Health Effects and Exposure Science, Pacific Northwest National Laboratory, Richland, WA 99352, USA; jt@pnnl.gov; 5 PETA International Science Consortium Ltd., London N1 9RL, UK; monitas@piscltd.org.uk; 6 Department of Chemistry, University of Fribourg, Chemin du Musée 9, 1700 Fribourg, Switzerland

**Keywords:** adherent cell cultures, carbon nanotubes, depletion, quantification, sedimentation, UV-VIS-NIR spectroscopy

## Abstract

The overt hazard of carbon nanotubes (CNTs) is often assessed using in vitro methods, but determining a dose–response relationship is still a challenge due to the analytical difficulty of quantifying the dose delivered to cells. An approach to accurately quantify CNT doses for submerged in vitro adherent cell culture systems using UV-VIS-near-infrared (NIR) spectroscopy is provided here. Two types of multi-walled CNTs (MWCNTs), Mitsui-7 and Nanocyl, which are dispersed in protein rich cell culture media, are studied as tested materials. Post 48 h of CNT incubation, the cellular fractions are subjected to microwave-assisted acid digestion/oxidation treatment, which eliminates biological matrix interference and improves CNT colloidal stability. The retrieved oxidized CNTs are analyzed and quantified using UV-VIS-NIR spectroscopy. In vitro imaging and quantification data in the presence of human lung epithelial cells (A549) confirm that up to 85% of Mitsui-7 and 48% for Nanocyl sediment interact (either through internalization or adherence) with cells during the 48 h of incubation. This finding is further confirmed using a sedimentation approach to estimate the delivered dose by measuring the depletion profile of the CNTs.

## 1. Introduction

The advanced physical and mechanical characteristics offered by carbon nanotubes (CNTs), such as high aspect ratio, excellent strength, and good conductivity, have increased their demand in the global market [1]. However, their growing use in commercial products and demonstrated biopersistence has raised concerns about their biological reactivity and potential health hazards. In vitro testing systems can be used to efficiently assess the toxicological potential of nanomaterials, but the use of toxicologically-irrelevant test concentrations decreases the relevance of the observed outcomes [2]. In particular, the in vitro cellular responses upon introduction of nanomaterials are often reported as a function of the administered dose, not of the actual dose of nanomaterials which are interacting with (or internalized by) cells [3]. Furthermore, in order for in vitro studies to be useful in predicting human outcomes in a regulatory context, the results must be converted to concentrations that would be relevant to human exposure [4]. Hence, it is critical that the in vitro concentrations of nanomaterials and their respective doses can be measured and/or modeled after scenarios relevant to potential human exposure (i.e., defined occupational exposure limits).

A few models have been developed to estimate in vitro concentration for various nanomaterials: the in vitro sedimentation, diffusion, and dosimetry (ISDD) model developed by Hinderliter and colleagues; the distorted grid (DG) model developed by Demokritou and colleagues; and the 3D-sedimentation-diffusion-dosimetry (3DSDD) model developed by Böhmert and colleagues estimate the deposited nanomaterial dose by measuring the effective density of nano-agglomerates in suspension or by taking into account the 3D distribution of cells in in vitro cell culture dishes [5,6,7]. However, these models were developed and are suitable only for low-aspect-ratio particles (e.g., spherical silica, gold, and metal oxide nanoparticles) and have a limited applicability for high-aspect-ratio materials (e.g., fibers such as CNTs) due to their different form factor and heterogeneity.

Herein, a simple approach is presented to quantify the delivered dose of multi-walled CNTs (MWCNTs) dispersed in protein-rich cell culture media (CCM) in the presence of epithelial cells cultured on the bottom of a cell culture well. This approach is based on the UV-VIS-near infrared (NIR) spectroscopy measurement of the extinction spectra of CNTs extracted from the cells after 48 h of incubation, allowing accurate determination of CNT concentrations inside or adhered to the cells. The results are compared to a sedimentation approach [8] based on the measurement of a CNT depletion profile (i.e., reduction of concentration over time) in the cell culture medium by UV-VIS-NIR.

We conclude that the developed approach provides precise estimates of CNT doses delivered to cells and will be very valuable for the effective use of in vitro systems in the assessment of the hazard posed by CNTs to human cells.

## 2. Materials and Methods

### 2.1. Sample Preparation

Mitsui-7 MWCNTs (Mitsui & Co., Ltd., Tokyo, Japan) were kindly provided by Professor Vicki Stone, Heriot-Watt University, Edinburgh, UK, while Nanocyl-7000 MWCNTs were obtained from the European Commission Joint Research Centre, ISPRA, Italy (originated from Nanocyl S.A., Sambreville, Belgium) and used without any further purification. Fifty µg/mL of CNT stock solution was stabilized in 1 mg/mL bovine serum albumin (BSA) (Sigma Aldrich, Darmstadt, Germany) in sterile Milli-Q water and the solution was sonicated for 3 h using an Elmasonic P30H cleaning unit, 100 W, 37 kHz, 30% (Elma Schmidbauer GmbH, Singen, Germany) with continuous horizontal shaking and water cooling [9]. The stock solution was redispersed in CCM containing 88% Rosewell Park Memorial Institute (RPMI, Gibco, Grand Island, NY, USA) 1640 medium supplemented with 10% fetal bovine serum (FBS; Gibco, Grand Island, NY, USA), 1% L-glutamine (200 mM, L-Glut; Life Technologies, Zug, Switzerland) and 1% penicillin/streptomycin (10,000 units/mL/10,000 μg/mL, Gibco, Grand Island, NY, USA) at 10 and 20 µg/mL.

### 2.2. Characterization Methods

Both types of CNTs were analyzed by means of TEM. CNT suspensions in water and CCM were spin-coated and dried on carbon-film square-mesh copper grids (Electron Microscopy Sciences, CF-300-Cu, Hatfield, PA, USA). TEM micrographs were taken with a Tecnai Spirit transmission electron microscope (FEI, Frankfurt, Germany) operating at 120 kV. Images were recorded at a resolution of 2048 × 2048 pixels (Veleta CCD camera, Olympus, Volketswil, Switzerland). The size and morphology of CNT dispersions were characterized by analyzing TEM micrographs using ImageJ (NIH, Bethesda, MD, USA). A total of 145 objects were subjected to quantification. The surface charge of the CNTs in CCM was determined by measuring the zeta potential at 25 °C using a phase amplitude light scattering (PALS) zeta potential analyzer (Brookhaven Instruments Corporation, Hotsville, NY, USA). A suspension of 10 µg/mL CNTs in CCM was incubated for 1 h and 48 h. Then, the sample was diluted five-fold to reduce the salt concentration, as this can affect the zeta potential measurement [10]. The Smoluchowski approximation was fitted to 10 cycles of electrophoretic mobility (EPM) measurement and 10 measurements were obtained for each sample to estimate the mean and standard deviation (SD). Hydrodynamic radii were measured by depolarized dynamic light scattering (DDLS) and the values have been provided as intensity-weighted size distributions as previously reported by Geers et al [11]. Briefly, samples were diluted into CCM and measurements were carried out at 37 °C with a scattering angle of 30°. DDLS spectra were collected using a commercial goniometer instrument (LS instruments AG, Fribourg, Switzerland) equipped with a 500 mW diode pumped solid-state laser (660 nm). Data was acquired over 15 min and five independent correlation functions were measured at 1 h and 48 h.

### 2.3. Fluorescence-Enhanced Dark Field Microscopy

Human alveolar type II epithelial cells (A549) were purchased from American Type Culture Collection (ATCC, Manassas, VA, USA) and cultured in CCM at 37 °C and 5% CO_2_ for 48–72 h until 80–90% cell confluency was reached. The cells were trypsinized and 20,000 cells were seeded into Falcon^®^8 well chamber slides (surface area per well = 0.7 cm^2^, Corning Inc., Corning, NY, USA) and grown overnight. Next, 750 µL of 10 or 20 µg/mL of Mitsui-7 or Nanocyl in CCM was added and the cells were incubated for 48 h. The cells were washed three times with phosphate-buffered saline (PBS) to remove any non-interacting CNTs. The cell monolayer was fixed with 4% paraformaldehyde (PFA) in PBS, followed by immunofluorescence staining. The cells were washed twice with PBS and rinsed in 0.1% Triton X-100 (Sigma Aldrich, Germany) in PBS for 5 min and subsequently in 1% BSA (Sigma Aldrich) in PBS for another 20 min. The cell layer was subsequently stained with rhodamine phalloidin (Invitrogen, Carlsbad, CA, USA) and 4’,6-diamidino-2-phenylindole, dihydrochloride (DAPI) for visualization of cellular F-actin and cell nuclei, respectively, for 20 min in the dark at room temperature, followed by washing with PBS. Cover slips were mounted onto glass slides for microscopy. The samples were visualized using a 100× objective lens and numerical aperture 0.8 for enhanced-darkfield (eDF) and numerical aperture 1.3 for fluorescence in a Cytoviva dual mode fluorescence-enhanced darkfield microscopy setup (Cytoviva Inc., Auburn, AL, USA). Three-dimensional image reconstructions were performed using Imaris software (Bitplane, Zurich, Switzerland).

### 2.4. CNT Cellular Uptake/Association Quantification by UV-VIS-NIR Spectroscopy

200,000 A549 cells were cultured in 12-well plates until they formed confluent cell monolayers (surface area = 3.8 cm^2^). Old media were removed and 1.5 mL of 10 or 20 µg/mL of Mitsui-7 or Nanocyl dispersed in fresh CCM was added and the cells were incubated for 48 h. The cells were washed three times with PBS to remove the excess of non-interacting CNTs before fixation with 4% PFA in PBS was performed. The washing process was repeated twice and 1 mL of fresh PBS was added gently onto the cells. The cell–CNT mixtures were then subjected to microwave-assisted digestion/oxidation [12]. Briefly, PBS buffer was removed and the cells were treated with a mixture 2:1 (*v*/*v*) of concentrated HNO_3_ (69%) and hydrogen peroxide (H_2_O_2_, 30%) (400 µL:200 µL). The cell layer was detached from the well plate by scraping with a micropipette and collected in certified 5 mL glass microwave vessels (Anton Paar, Graz, Austria). Microwave-assisted digestion was performed with a maximum temperature and pressure of 140 °C and 20 bar, respectively. The microwave heating protocol was programmed as follows: (i) 100 W for 40 min (10 min ramp), (ii) 200 W for 20 min (10 min ramp), (iii) 300 W for 20 min (10 min ramp), (iv) 500 W for 20 min (10 min ramp), and (v) 0 W for 20 min for the cooling step. After cooling down to room temperature, the oxidized CNT samples were diluted with 2 mL of 1 M sodium hydroxide (NaOH) to stabilize the CNTs and trapped in a filter membrane by filtering them under vacuum using a 0.22 µm hydrophilic polyvinylidene fluoride (PVDF) membrane (Durapore^®^, Millipore, Cork, Ireland). The CNTs were released from the membrane filters by sonication in 1 mL of Milli-Q water for 30 min. The hydrophilic property of PVDF membranes exhibits a reliable chemical stability even at the harsh conditions to which the membranes were exposed, confirming that during the sonication step, only the CNTs were recollected. The retrieved samples were then placed in a 10 mm path length disposable UV cuvette (Brand^®^, Brand GmbH, Wertheim, Germany; wavelength range: 230 to 900 nm) and refilled with Milli-Q water to a maximum volume of 2 mL. The extinction spectra of the CNTs were measured using a JASCO V-670 UV-VIS-NIR spectrophotometer. The position of the beam, which had a width of 6.4 mm and a height of 15.1 mm, related to the cuvette was centered at 11.6 mm from the bottom of the cuvette. In order to build a calibration curve, a 500 µL aliquot of CNT suspension (25 µg/mL in CCM) was subjected to the same procedure described above. A series of dilutions of oxidized CNT suspension were carried out to obtain standards within a concentration range of 0.06 to 2.48 µg/mL for Mitsui-7 and 0.12 to 2.63 µg/mL for Nanocyl. Two milliliters of these standards were placed in a cuvette and the corresponding extinction spectrum was acquired. Extinction values were obtained by integrating the absorbance corresponding to the wavelength range from 640 to 990 nm from the extinction spectra and plotting it against the known concentration of the dilution. The concentration of the cellular uptake/association and remaining suspended fraction in three independent replicates were employed for establishing accuracy of the method. The results have been expressed as mean absolute recovery. Precision of the method has subsequently been expressed as coefficients of variation (% CVs).

### 2.5. Monitoring CNT Sedimentation Profile by UV-VIS-NIR Spectroscopy

The extinction spectral evolution of CNTs in CCM over 48 h at 37 °C was monitored using a JASCO V-670 UV-Vis-NIR spectrophotometer. Two milliliters of Mitsui-7 or Nanocyl dispersed in CCM at concentrations of 10 and 20 µg/mL were introduced into a 10 mm path length disposable UV cuvette (Brand^®^; wavelength range: 230 to 900 nm; area = 1 cm^2^) and placed in the thermostatic cell holder of the spectrophotometer set to 37 °C. Baseline correction was carried out using Milli-Q water. To avoid any interference with proteins (λ_absorbance_ = 413 nm) and phenol red (λ_absorbance_ = 558 nm) present in the CCM, a 640 to 900 nm analysis window was selected. The amount of CNTs remaining in suspension (*[CNTs]_suspension_*) over 48 h was determined using a calibration curve based on the Beer-Lambert law obtained by plotting a known concentration of CNTs against the average optical extinction from 640 to 900 nm. In order to confirm that the Beer-Lambert law was fulfilled in the range of the studied concentrations, a series of dilutions in CCM were carried out to obtain final concentrations between 0 to 24 µg/mL for Mitsui-7 and 0 to 20 µg/mL for Nanocyl. The extinction spectra of 2 mL of these standards were acquired as described above with the extinction values integrated and plotted against the known concentration of suspended CNTs. The amount of CNTs deposited at the bottom of the cuvette, i.e., the dose *([CNTs]_D_*), was estimated using the mass balance Equation (1), i.e.,

*[CNTs]_D_ = [CNTs]_initial_ − [CNTs]_suspension_* (1)

where *[CNTs]_initial_* corresponds to the initial concentration of CNTs. The estimated dose of CNTs, *Dose_D_*, was calculated as *[CNTs]_D_* divided by the area of the cuvette and expressed in µg/cm^2^.

## 3. Results and Discussion

### 3.1. Characterization of CNTs

**Figure 1:**
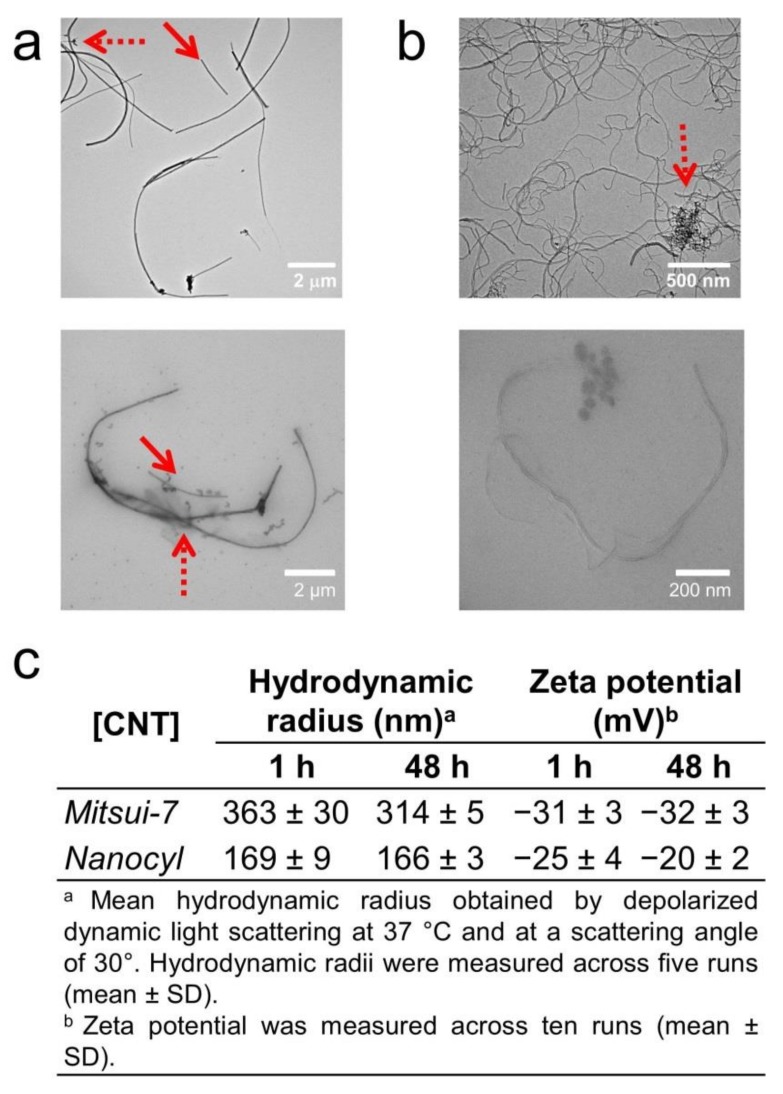
Characterization of carbon nanotubes (CNTs). TEM images of (**a**) Mitsui-7 and (**b**) Nanocyl dispersions in water (top) and cell culture medium (CCM) (bottom) showing their polydispersity and structural heterogeneity (i.e., single and agglomerate). The round particles observed are salts, which are present in CCM. Solid and dashed arrows show single CNTs and agglomerates of CNTs, respectively. (**c**) Hydrodynamic radius and zeta potential of the two types of CNTs in CCM post 1 and 48 h of incubation in CCM.

Two types of MWCNTs, namely, Mitsui-7 CNTs (or simply Mitsui-7) and Nanocyl-7000 CNTs (or simply Nanocyl) were used, since they possess different physico-chemical characteristics [13]. The powder was dispersed and stabilized with BSA [14,15,16,17,18] in water at a concentration of 50 µg/mL as described by the Nanoreg ECOTOX SOP [9] (i.e., the protocol for producing reproducible dispersions of manufactured nanomaterials in environmental exposure media). The size and morphology of CNT dispersions were characterized by TEM both in water and CCM (Figure 1a,b and Figures S1–S4). The CNTs showed different aspect ratios: 89 for Mitsui-7 with diameter × length (d × L) 66 nm × 5.9 µm (Figure 1a and Figure S1) and 33 for Nanocyl with diameter × length 14 nm × 469 nm (Figure 1b and Figure S2). 

These TEM images also show that the CNT dispersions contain both single CNTs and small agglomerates (pointed out by red arrows in Figure 1). These CNTs were further diluted in CCM at the administered concentrations of 10 and 20 µg/mL, which are known not to alter their stability [19]. Their corresponding TEM micrographs are shown in Figures S3 and S4, respectively. Depolarized dynamic light scattering (DDLS) and zeta potential measurements show CNT hydrodynamic radii in CCM of around 363 nm (Mitsui-7) and 170 nm (Nanocyl) with zeta potentials of −30 mV and −25 mV, respectively. No significant change of hydrodynamic radii nor zeta potential was observed over a longer period of time (e.g., 48 h, see Figure 1c), indicating the stability of BSA-coated CNTs.

### 3.2. Interaction of CNTs with Cells

**Figure 2:**
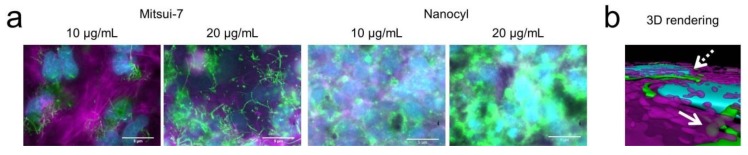
(**a**) Enhanced darkfield fluorescence images of human lung epithelial cells (A549) after 48 h of exposure to 10 and 20 µg/mL of Mitsui-7 (green, left panel) or Nanocyl (green, right panel). (**b**) 3D render of z-stack images show the presence of internalized (solid arrow) and strongly adherent Mitsui-7 on the outer cell membranes of the cells (dashed arrow). The F-actin cytoskeletons (magenta) and cell nuclei (cyan) were counterstained with rhodamine phalloidin and 4’,6-diamidino-2-phenylindole, dihydrochloride (DAPI), respectively. The 3D rendering was performed using Imaris software (Bitplane AG, Zurich, Switzerland). Scale bars = 5 µm.

To address the biological interaction between CNTs and cells, in vitro cellular uptake and imaging experiments were conducted using human alveolar type II epithelial cells (A549) which form a confluent cell layer covering the complete surface of the plastic well. Alveolar epithelial cells are one of the first target cells when CNTs are inhaled [20] and previous in vitro and in vivo data have demonstrated that CNTs inside the lungs are not only incorporated into alveolar macrophages [21,22] but also by epithelial cells, including type I [23] and type II epithelial cells [13,24,25,26,27]. In addition, A549 exposure to CNTs such as Mitsui-7 and Nanocyl at concentrations of up to 20 µg/mL did not result in any cytotoxic response [13]. Briefly, A549 were cultured in the presence of both CNTs at concentrations of 10 and 20 µg/mL for 48 h. Next, the cells were washed three times with PBS to remove non-interacting CNTs. Using fluorescence and an eDF microscope [24], the presence of Mitsui-7 and Nanocyl associated with A549 cells was shown. A549 cells were confluent on the glass slide (Figure S5). Imaging data and 3D rendered images show the presence of internalized and strongly adherent CNTs on the outer cell membranes of the cells (Figure 2a,b). Due to the larger size and higher aspect ratio of Mitsui-7, both single CNTs and agglomerates could be visualized, while Nanocyl could be only observed as agglomerates; smaller or single Nanocyl CNTs could not be detected with this method. As expected, the number of both CNTs on the outer cell membranes increased when the exposure concentration of CNTs was increased from 10 to 20 µg/mL (Figure 2a).

### 3.3. Sample Preparation by Extraction of Cell-Associated CNTs and Determination of Concentration by UV-VIS-NIR Spectroscopy

**Figure 3:**
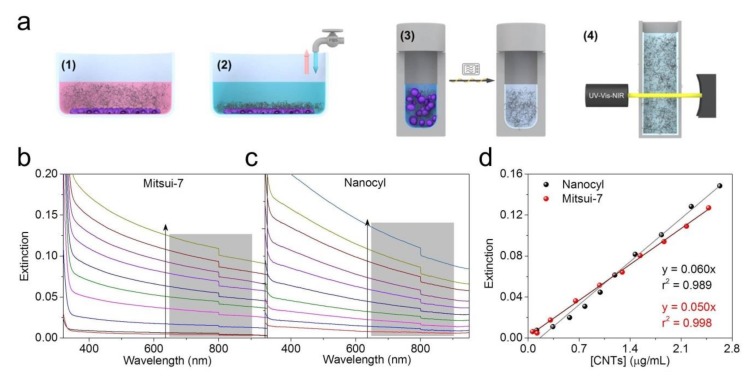
(**a**) A schematic illustration of the experimental steps used to determine the dose of CNTs attached to and/or internalized by A549 cells. The steps include (1) incubation of CNTs, (2) the fixation and washing process, (3) sample preparation by microwave-assisted acid digestion and oxidation treatment, and (4) CNT quantification by UV-VIS-NIR spectroscopy. Extinction spectra of purified and oxidized CNTs at different concentrations in the range of (**b**) 0.06 to 2.48 µg/mL for Mitsui-7 and (**c**) 0.12 to 2.63 µg/mL for Nanocyl after microwave-assisted acid digestion. (**d**) Calibration curve of oxidized CNTs (O-CNTs). Extinction values for the ten-point dilution series of oxidized and purified CNT samples shown in (b) and (c) have been integrated from 640 to 900 nm. A linear relationship (black and red lines) between the concentration of CNTs and the extinction was found for the range of concentrations studied here.

Quantification of CNTs in complex biological media based on the CNTs’ optical extinction properties still possesses some fundamental issues. Several studies have demonstrated that the components of CCM could influence the optical response of the CNTs, leading to an under- or over-estimation of the obtained dosimetry values [28,29]. In addition, the optical extinction properties of CNT suspensions could also be affected by the presence of dispersants [30]. Therefore, in this study, an experimental strategy was designed to measure the in vitro CNT dose by eliminating matrix interferences (see scheme in Figure 3a). Briefly, A549 epithelial cell monolayers (total cell area = 3.8 cm^2^) were exposed to both CNTs at concentrations of 10 and 20 µg/mL for 48 h (Figure 3a, step 1). The cell layers were then washed to remove non-adherent CNTs (Figure 3a, step 2). After this, the samples were subjected to a microwave-assisted sampling method to extract CNTs (Figure 3a, step 3) by destroying the organic components. Microwave-assisted acid digestion is a well-established method used to destroy, among other things, plasma, tissues, and blood with high efficiency [31]. As a consequence of this analytical treatment, the CNTs become oxidized (becoming O-CNTs), resulting in an increase of their colloidal stability [12]. Oxidized and purified CNTs were then redispersed in water and their optical extinction was recorded using UV-VIS-NIR spectroscopy as the detection method (Figure 3a, step 4). Nanocyl and Mitsui-7 suspensions in CCM at a concentration of 25 µg/mL were subjected to the same treatment and used to perform a calibration curve.

The microwave-assisted acid digestion/oxidation treatment resulted in well-dispersed, oxidized CNTs independently of the type of CNT, enabling the accurate quantification of the cell-associated CNTs (referred to here as measured *Dose_D_*) without any interference from the matrix interference/environment (see spectra sets in Figure 3b for Mitsui-7 and Figure 3c for Nanocyl). The extinction coefficients obtained for the calibration curves, namely, 0.050 mL·cm^−1^·µg^−1^ for Mitsui-7 and 0.060 mL·cm^−1^·µg^−1^ for Nanocyl (Figure 3d), are in good agreement with those previously reported in the literature for a well-dispersed CNT suspension [32]. The method used also showed high and consistent recoveries for both concentrations; for 10 µg/mL the recovery was 95% for Mitsui-7 and 93% for Nanocyl while for 20 µg/mL the recovery was 88% for Mitsui-7 and 68% for Nanocyl.

Table 1 shows the cell-associated CNT dose as measured by UV-VIS-NIR spectroscopy after sampling (i.e., post-48 h incubation for both CNT and administered doses). As expected, an increase in the cell-associated CNT dose with increasing initial CNT concentrations was observed in both cases. Interestingly, our result shows a difference in maximum measured *Dose_D_* (or percentage of delivered fraction) for both CNTs after 48 h: 3.33 µg/cm^2^ (85%) and 6.44 µg/cm^2^ (82%) for Mitsui-7 (for 10 and 20 µg/mL) and 1.88 µg/cm^2^ (48%) and 2.41 µg/cm^2^ (31%) for Nanocyl (for 10 and 20 µg/mL), respectively. We hypothesize that the higher delivery of Mitsui-7 in comparison to Nanocyl can be attributed to its larger size and aspect ratio, as both sedimentation and diffusion toward the cell surface is likely to take place, while only diffusion is responsible for the delivery of Nanocyl.

### 3.4. Complementary Study Using Sedimentation Approach

**Figure 4:**
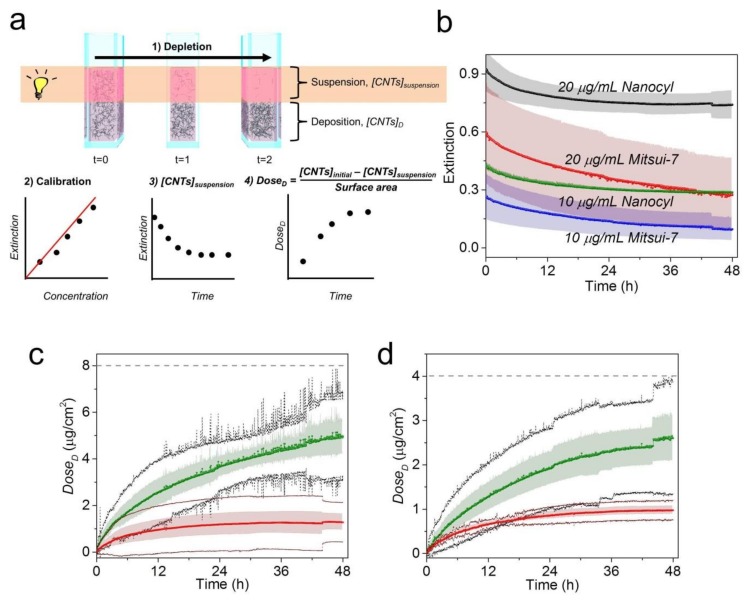
(**a**) Illustrative scheme of the experimental design used to monitor the CNT depletion profile and estimation of deposited dose (*Dose_D_*) using the Beer-Lambert law. The approach consists of four steps: (1) monitoring the CNT depletion in real time over 48 h, (2) creating a calibration curve, (3) determining the concentration of CNTs remaining in the suspension (*[CNTs]_suspension_*) using the calibration curve and the Beer-Lambert law equation (see Figure S6), and (4) estimating the deposited dose (estimated *Dose_D_*) using the mass balance equation. (**b**) Depletion profiles obtained by integration of the extinction for the spectral window 640–900 nm for Nanocyl and Mitsui-7 in CCM over 48 h for two concentrations, i.e., 20 (black dots for Nanocyl and red dots for Mitsui-7) and 10 µg/mL (green dots for Nanocyl and blue dots for Mitsui-7), respectively. The shadowed area represents the standard deviation (SD) of three independent repetitions. A comparison between estimated *Dose_D_* of Mitsui-7 (green) and Nanocyl (red) at a concentration of (**c**) 20 and (**d**) 10 µg/mL is given. The shadowed area represents the SD of three independent repetitions. Black and dark red dots show 95% confidence limits for Mitsui-7 and Nanocyl, respectively. The dashed lines represent here the initial dose of CNTs (*[CNTs]_Initial_*/surface area) expressed in µg/cm^2^: 7.9 and 3.9 µg/cm^2^ for 20 and 10 µg/mL, respectively. The dose corresponds to a cell growth area of 3.8 cm^2^.

Due to the absence of any standard technique able to verify our result, we followed a sedimentation approach developed by Thomas et al., Cho et al., Rischitor et al., and Spyrogianni et al. [8,33,34,35] which has been successfully used for the quantification of concentrations of spherical nanomaterials such as gold nanoparticles. In this regard, the dose of nanoparticles arriving at the bottom of the cell culture wells (referred to here as estimated *Dose_D_*) is calculated through monitoring of the decrease in particle concentration (i.e., depletion profile) in the upper part of suspension. It is important to note that this approach assumes that all sedimented particles will interact with cells and are considered in the dose calculation; therefore, no cells are needed in the design of the experiment. Owing to the stability of CNTs, which do not change over 48 h (Figure 1c), it is therefore possible to study the CNTs’ depletion profile in CCM by means of UV-VIS-NIR spectroscopy. The procedure is shown schematically in Figure 4a (step 1). The variation in optical extinction in real time over 48 h for both CNTs in CCM was monitored within the upper part of the suspension. Figure S6 shows the spectra set for Mitsui-7 and Nanocyl in which can be clearly observed the decay of optical extinction in both CNTs at the two concentrations, as well as the matrix interferences in the overall CNT-CCM spectra, which include BSA (λ_absorbance_ = 280 nm), proteins (λ_absorbance_ = 413 nm), and phenol red (λ_absorbance_ = 558 nm). Due to the presence of matrix interference, the depletion profile was determined by integration of the spectral window from 640–900 nm (Figure 4b). The extinction values of the depletion profile were then converted on deposited doses of CNTs using Equation (1) (Figure 4a(2–4)). Prior results have confirmed that there is a linear relationship between the optical extinction and concentration of CNTs (Figure S7) and that the extinction coefficients (ε) of CNTs are nearly independent of the wavelength because the CNT concentration range is very low [36]. It should be also pointed out that the absorbance efficiency of single MWCNTs is much greater than the scattering efficiency [37], consequently meaning that the influence of scattering in a well-dispersed and diluted (<1 mg/mL) CNT suspension should be minimal, allowing the utilization of the Beer-Lambert law for determining the suspended CNT concentrations and dose quantification.

A detectable difference was observed in the estimated *Dose_D_* profiles of Mitsui-7 and Nanocyl for both administered concentrations (Figure 4c for 20 µg/mL and Figure 4d for 10 µg/mL). Specifically, the estimated *Dose_D_* values at 48 h for Mitsui-7 (2.60 and 4.98 µg/cm^2^) show a 2.7-fold and 4.0-fold higher increase than those of Nanocyl (0.98 and 1.28 µg/cm^2^) at 10 and 20 µg/mL concentrations, respectively (Table S1). Considering that the estimated *Dose_D_* profiles are dependent on concentration and the CNT aspect ratio, a higher dose for Mitsui-7 than Nanocyl at all measured time points is expected due to the different settling/diffusion tendencies toward the bottom of a cuvette (in the case of the cell experiment: the bottom of the cell culture plate).

The within-sample coefficient of variation (CV) (i.e., the standard deviation divided by the mean) was found to range from 3% to 52% and 11% to 34% for Nanocyl and Mitsui-7, respectively (Table S1). CVs greater than 20% can be associated with a variation in low CNT dose (specifically for the case of Nanocyl at 20 µg/mL) which approaches the method’s limit of detection, as well as the heterogeneity of the materials [38].

The estimated dose values showed the same trend with respect to the measured *Dose_D_* obtained by microwave-assisted digestion (see the specific values in Table 1 and deposition profiles in Figure 2); however, the estimated values were found to be lower (i.e., underestimated). This difference could be attributed not only to the matrix interferences explained above but also to the difference in agglomeration behavior in the presence and absence of cells modifying the sedimentation rate of CNTs. The presence of agglomeration of nanomaterials in vitro induced by cell debris or large proteins secreted by cells has been previously observed [39]. In the case of in vitro measurement, higher doses are expected as agglomeration accelerates sedimentation and increases the concentration of CNTs interacting with cells.

Moreover, the difference in the geometry of the sample holder (i.e., filling height) used in the two approaches could also be responsible for higher values in the measured *Dose_D_* in comparison to that which is estimated. Even though a similar volume of initial CNTs were used, the microwave-assisted approach used 12-well plates (filling height = 0.39 cm) while the sedimentation approach used plastic cuvettes (filling height = 2 cm). This difference would have influenced the settling time of the CNTs due to the increase in the required travel distance toward the bottom of the cuvette with respect to the well-plate. However, it is difficult to overcome this limitation and for a more comprehensive understanding of CNT behavior in biological systems, it is recommended that two different approaches for one endpoint be used [40].

Nevertheless, both approaches clearly demonstrated that Mitsui-7 has a two- to three-fold higher cell-associated dose than Nanocyl after 48 h. Apart from the difference in their stiffness, this outcome (i.e., higher dose) can indeed provide a mechanistic view of the differences in biological behaviors, e.g., earlier onset of inflammation and DNA damage, fibrosis, and a unique fibrotic gene expression profile which is induced by large size CNTs similar to Mitsui-7 in comparison with smaller ones (i.e., Nanocyl), as previously reported by Poulsen et al [41].

## 4. Conclusions

In conclusion, in this work we have demonstrated approaches based on UV-VIS-NIR spectroscopy to quantify MWCNTs suspended in CCM for adherent cell cultures. The approaches consisted of direct in vitro measurement and estimation using sedimentation, and are independent of the CNT types, dispersant (e.g., BSA), and the colloidal stability of the materials in CCM. Our findings show a higher delivered dose of Mitsui-7 in comparison to Nanocyl post 48 h incubation to A549 cells, which can be attributed to the higher aspect ratio profile of Mitsui-7. It is important to emphasize that there was a slight underestimation of the estimated dose (estimated *Dose_D_*) in comparison to direct measurement (measured *Dose_D_*), which we hypothesize is due to the presence/absence of cells and the geometry of well plates/cuvettes (i.e., difference in filling height). Nevertheless, the hazard assessment of any nanomaterials with in vitro testing platforms remains highly pertinent to the field, with the need to understand and determine specific effect-level concentrations becoming increasingly important in terms of establishing structure–activity and/or dose–response relationships. Thus, the approach presented herein for high-aspect-ratio carbon nanomaterials is a vital component towards this vision.

## Figures and Tables

**Table 1:** Comparison between measured *Dose_D_* from cell uptake experiments and estimated *Dose_D_* from sedimentation approach for Mitsui-7 and Nanocyl post 48 h of CNT incubation.

CNT Type; [CNTs]_Initial_ [µg/cm^2^]	Estimated *Dose_D_* [µg/cm^2^] ^a^	Delivered Fraction (%)	Measured *Dose_D_* [µg/cm^2^] ^a^	Delivered Fraction (%)
Mitsui-7; 3.9	2.60 ± 0.51	66 ± 11	3.33 ± 1.38	85 ± 35
Nanocyl; 3.9	0.98 ± 0.09	25 ± 2	1.88 ± 0.70	48 ± 18
Mitsui-7; 7.9	4.98 ± 0.76	63 ± 8	6.44 ± 2.03	82 ± 26
Nanocyl; 7.9	1.28 ± 0.34	16 ± 4	2.41 ± 0.94	31 ± 12

^a^ Number of independent samples per dose = 3. The doses corresponded to an area of measurement of 3.8 cm^2^.

## Supplementary Materials

The following are available online at https://www.mdpi.com/2079-4991/9/12/1765/s1, Figure S1: Representative TEM images of BSA-stabilized Mitsui-7 in H_2_O. Figure S2: Representative TEM images of BSA-stabilized Nanocyl in H_2_O. Figure S3: Representative TEM images of Mitsui-7 in CCM. Figure S4: Representative TEM images of Nanocyl in CCM. Figure S5: Enhanced darkfield-fluorescence image of A549 human lung epithelial cells cultured in CCM. Figure S6: Spectral evolution of the optical extinction of Mitsui-7 and Nanocyl. Figure S7: UV-VIS analysis of Mitsui-7 and Nanocyl. Table S1: Estimated values of CNT deposited doses at 4, 24, and 48 h.
