# Supplementary material for: Quantification of Carbon Nanotube Doses in Adherent Cell Culture Assays Using UV-VIS-NIR Spectroscopy
Source: Nanomaterials (Basel). 2019 Dec 11;9(12):1765. doi: 10.3390/nano9121765 (PMC6956054; doi:10.3390/nano9121765)
Supplement: Supplementary file 1 [file nanomaterials-09-01765-s001.pdf]

Supporting Information

Quantification of Carbon Nanotube Doses in Adherent Cell Culture Assays Using UV-VIS-NIR Spectroscopy

Dedy Septiadi [1,†], Laura Rodriguez-Lorenzo [1,2,†], Sandor Balog [1], Miguel Spuch-Calvar [1], Giovanni Spiaggia [1], Patricia Taladriz-Blanco [1], Hana Barosova [1], Savvina Chortarea [1], Martin J. D. Clift [3], Justin Teeguarden [4], Monita Sharma [5], Alke Petri-Fink [1,6,*] and Barbara Rothen-Rutishauser [1,*]

[1] Adolphe Merkle Institute, University of Fribourg, Chemin des Verdiers 4, 1700 Fribourg, Switzerland; dedy.septiadi@unifr.ch (D.S.); laura.rodriguez-lorenzo@inl.int (L.R.-L.); sandor.balog@unifr.ch (S.B.); miguel.spuch-calvar@unifr.ch (M.S.-C.); giovanni.spiaggia@unifr.ch (G.S.); patricia.taladrizblanco@unifr.ch (P.T.-B.); hana.barosova@unifr.ch (H.B.); savvina.chortarea@empa.ch (S.C.)

[2] Department of Life Sciences, Nano for Environment Unit, Water Quality Group, Av. Mestre José Veiga s/n, 4715-330 Braga, Portugal

[3] In Vitro Toxicology Group, Swansea University Medical School, SA2 8PP Swansea, Wales, UK; m.j.d.clift@swansea.ac.uk

[4] Health Effects and Exposure Science, Pacific Northwest National Laboratory, Richland, WA 99352, USA; jt@pnnl.gov

[5] PETA International Science Consortium Ltd., N1 9RL London, UK; monitas@piscltd.org.uk

[6] Department of Chemistry, University of Fribourg, Chemin du Musée 9, 1700 Fribourg, Switzerland

* Correspondence: alke.fink@unifr.ch (A.P.-F.); barbara.rothen@unifr.ch (B.R.-R.)

† These authors contributed equally to this work.

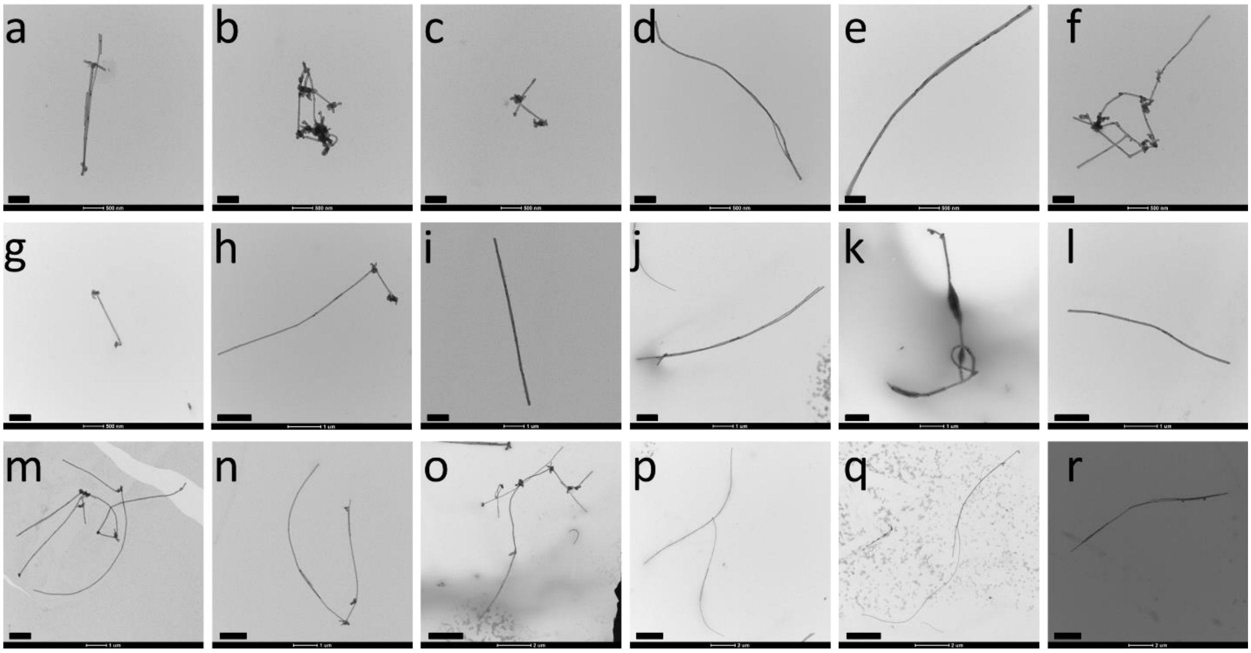

Figure S1. Representative TEM images of BSA-stabilized Mitsui-7 in H_2O. The scale bar for panel **a-g**. 500 nm, **h-n**. 1 μm and **o-r.** 2 μm. The CNT dispersion contains both single CNTs and small bundles.

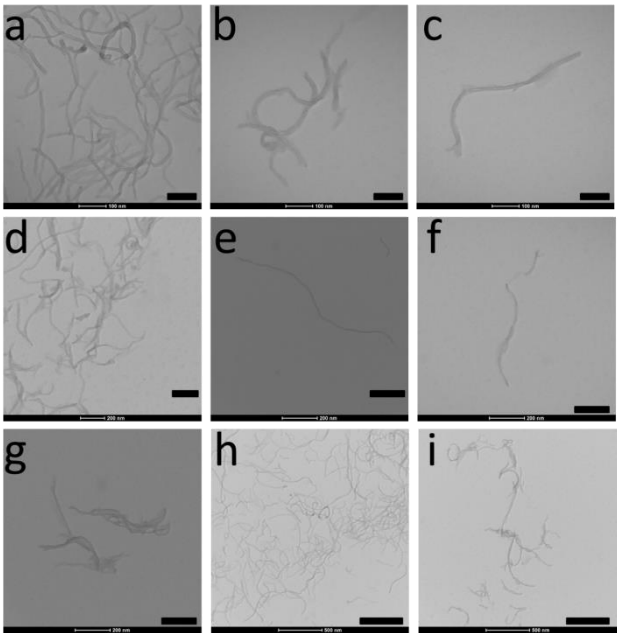

Figure S2. Representative TEM images of BSA-stabilized Nanocyl in H_2O. The scale bar for panel **a-c**. 100 nm and **d-i**. 200 nm.

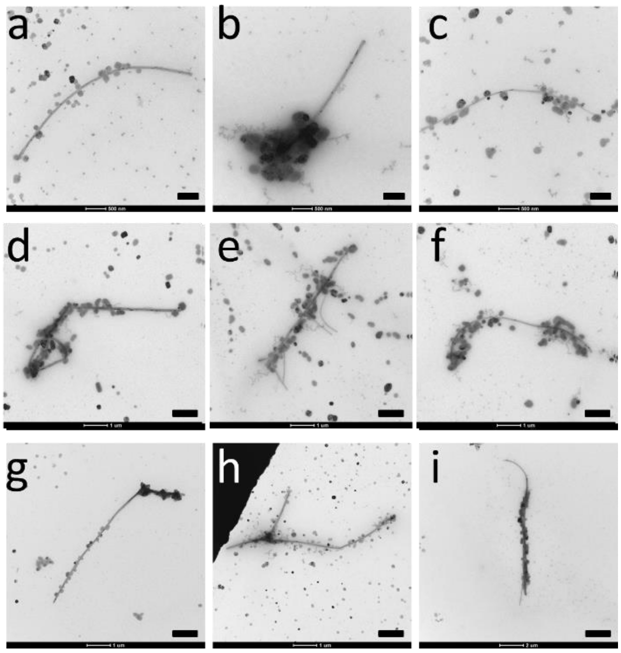

Figure S3. Representative TEM images of Mitsui-7 in CCM. Scale bar for panel **a-c**. 500 nm, **d-h**. 1 μm and **i**. 2 μm. Round particles observed are salts which are present in CCM.

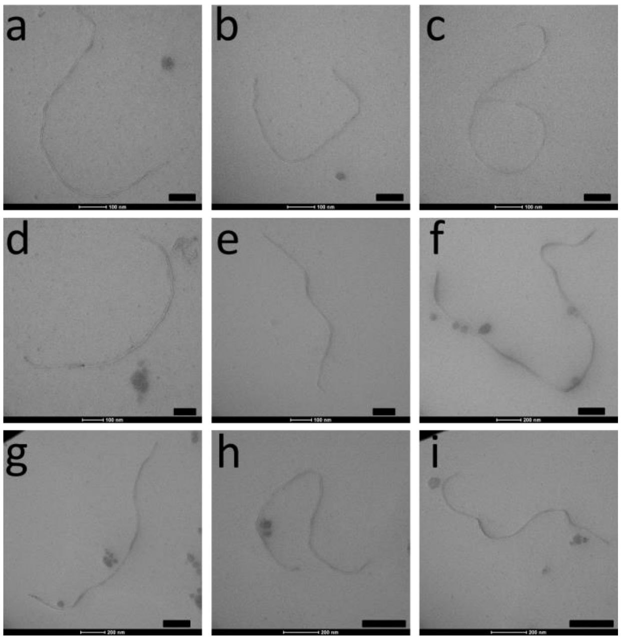

Figure S4. Representative TEM images of Nanocyl in CCM. Scale bar for panel **a-e**. 100 nm, and **f-1.** 200 nm.

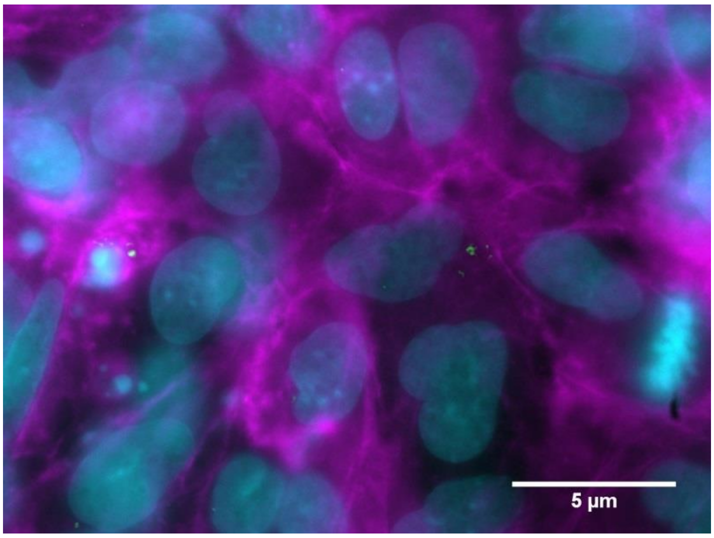

Figure S5. Enhanced darkfield-fluorescence image of A549 human lung epithelial cells cultured in CCM. The F-actin cytoskeleton (magenta) and cell nuclei (cyan) were stained with Rhodamine Phalloidin and DAPI respectively.

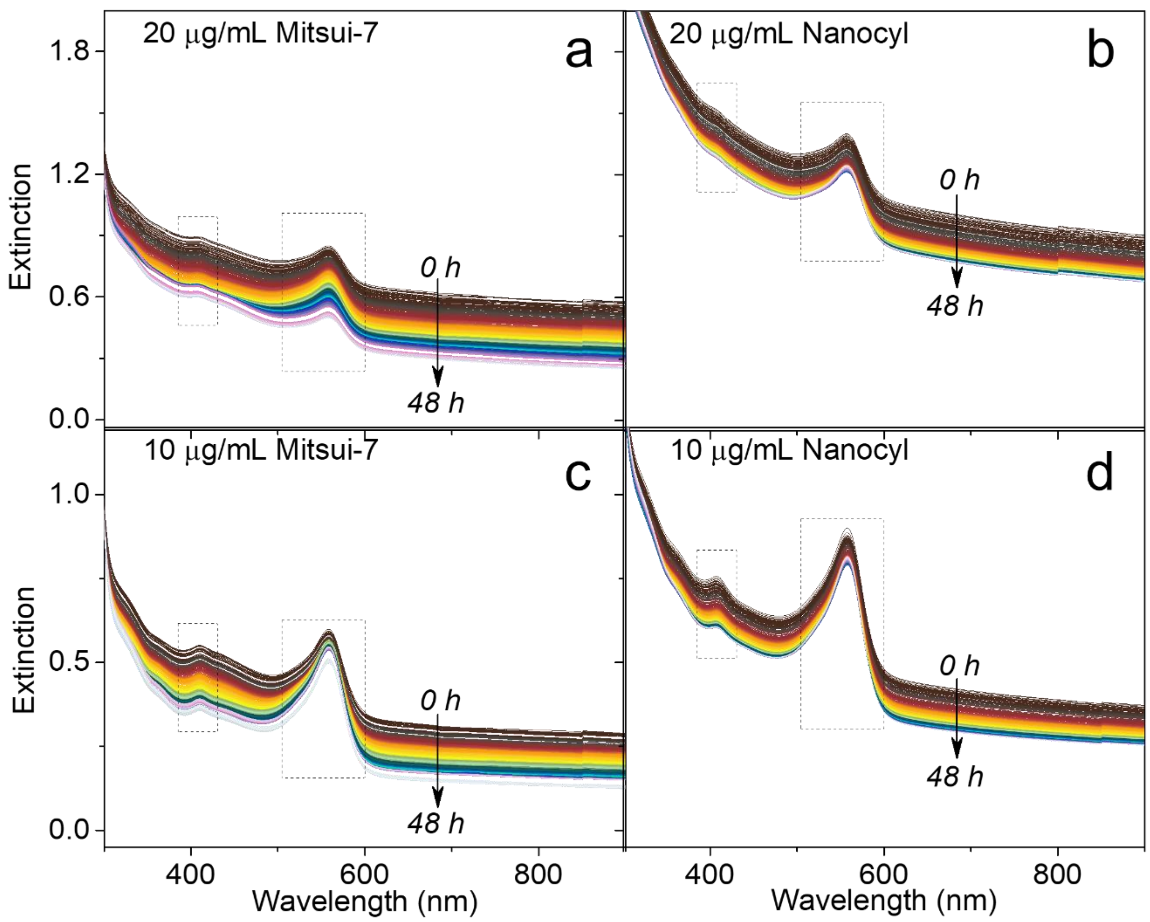

Figure S6. Spectral evolution of the optical extinction of Mitsui-7 and Nanocyl. Mitsui-7 and and Nanocyl are dispersed in CCM at initial concentration of 20 μg/mL (panel a and b) and 10 μg/mL (panel c and d) over 48 h at 37 °C. In all the cases, a decay of optical extinction (black arrow) is observed over time. The bands framed in dashed rectangles show clearly the matrix interferences from proteins (λ = 413 nm) and phenol red (λ = 558 nm).

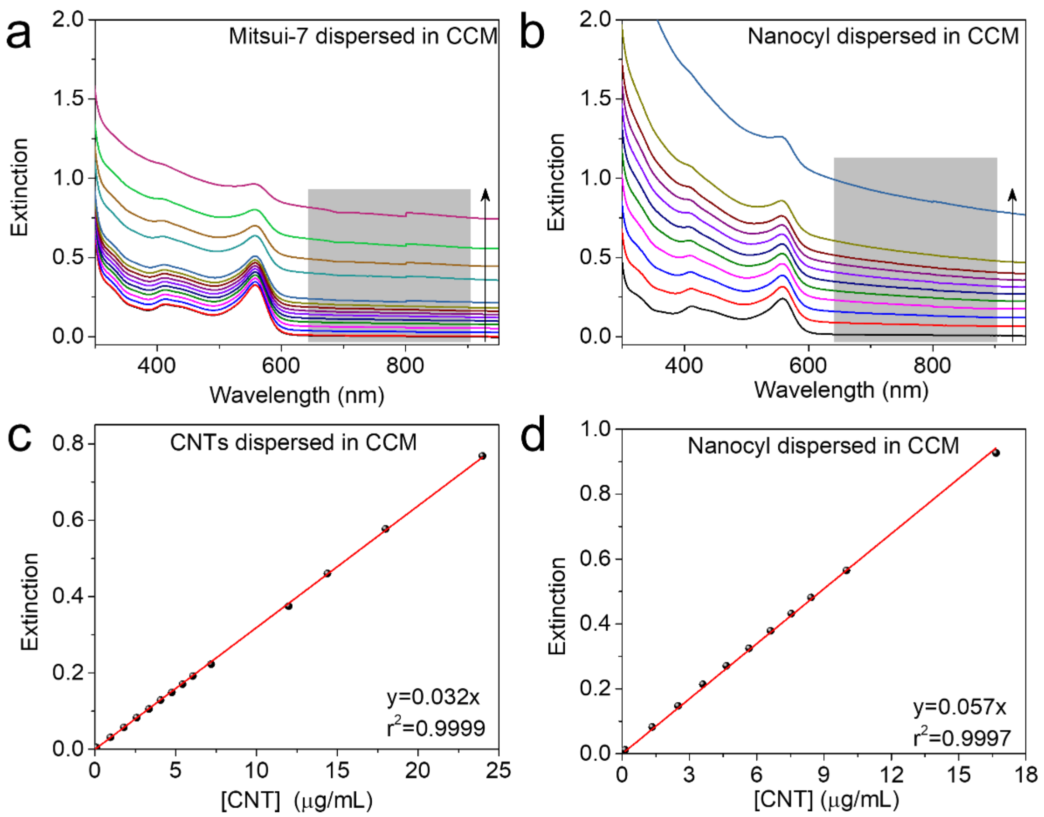

Figure S7. UV-VIS analysis of Mitsui-7 and Nanocyl. **a**. Mitsui-7 and **b**. Nanocyl are dispersed in CCM at different concentrations. Experimentally determined standard curves of **c**. Mitsui-7 and **d**. Nanocyl dispersions in CCM. Extinction values for a seventeen-point of Mitsui-7 samples and eleven-point of Nanocyl dilution series were measured using an UV-Vis-NIR spectrophotometer and integrated from 640 to 900 nm (indicated by grey square). A linear relationship (red line panel c and d) between the concentration of CNTs and the extinction was found at the range studied here.

Table S1. Estimated values of CNT deposited doses at 4, 24 and 48 h.

Dose$_0$ [μg/cm^2]	*Time point [h]*	*CCM*	
		Estimated Dose$_D$ [μg/cm^2][a] (Delivery fraction %)	*CV* [%][b]
Mitsui-7			
3.9	4	0.63 ± 0.22 (16 ± 4)	34
3.9	24	1.92 ± 0.36 (49 ± 7)	18
3.9	48	2.60 ± 0.51 (66 ± 11)	19
7.9	4	1.34 ± 0.33 (17 ± 3)	24
7.9	24	3.69 ± 0.42 (47 ± 4)	11
7.9	48	4.98 ± 0.76 (63 ± 8)	15
Nanocyl			
3.9	4	0.38 ± 0.01 (10 ± 1)	3
3.9	24	0.86 ± 0.08 (22 ± 2)	9
3.9	48	0.98 ± 0.09 (25 ± 2)	9
7.9	4	0.58 ± 0.30 (7 ± 4)	52
7.9	24	1.19 ± 0.46 (15 ± 6)	39
7.9	48	1.28 ± 0.34 (16 ± 4)	27

[a] Number of independent samples per dose and time point = 3 (mean ± SD). The dose corresponds to area of measurement of 3.8 cm^2.

[b] CV is defined as the SD divided by the mean, with the result reported as a percentage.
